# Data notes on the proteomics of *Dendrobium huoshanense* under pb treatment

**DOI:** 10.1186/s12863-024-01205-1

**Published:** 2024-02-21

**Authors:** Jun Dai, Yingyu Zhang, Yunpeng Zhang, Yan Wang, Xiaoyuan Ding, Cheng Song

**Affiliations:** 1 Anhui Engineering Research Center for Eco-agriculture of Traditional Chinese Medicine, College of Biological and Pharmaceutical Engineering, West Anhui University, 237012 Luan, China; 2 Endocrinology and Metabolism Center, The First Affiliated Hospital, College of Clinical Medicine of Henan, University of Science and Technology, 471003 Luoyang, China; 3 Sichuan Academy of Medical Sciences and Sichuan Provincial People’s Hospital, 610072 Chengdu, China

**Keywords:** Lead metal, *Dendrobium huoshanense*, Free radical burst, Antioxidant system, Abiotic stress

## Abstract

**Objectives:**

Pb stress has a negative impact on plant growth by interfering with photosynthesis and releasing reactive oxygen species, causing major risks such as heavy metal ion accumulation in the soil matrix. A proteomics experiment was conducted to determine whether protein levels of *Dendrobium huoshanense* changed in response to Pb stress seven to fifteen days after being sprayed with a 200 mg/L Pb (NO_3_)_2_ solution. The proteomic data we gathered provides a model for investigations into the mechanisms underlying Dendrobium plant resistance to heavy metal stress.

**Data description:**

A label-free quantitative proteomics approach was employed to examine the variations in protein expression levels of *D. huoshanense* at different times of Pb(NO_3_)_2_ treatment. We submitted the raw data obtained from these proteomics sequencing experiments to the ProteomeXchange database with the accession number PXD047050. 63,194 mass spectra in total were compared after being imported into the Proteome Discoverer software for database search. A total of 12,402 spectral peptides were identified with a confidence level exceeding 99%, which resulted in the identification of 2,449 significantly differential proteins. These proteins can be utilized for screening, functional annotation, and enrichment analysis of differentially expressed proteins before and after heavy metal treatment experiments.

## Objective

Heavy metals are a limiting factor that negatively impacts the growth and productivity of crops [1]. The accumulation of Pb causes stomatal closure and limits the performance of photosynthetic enzymes, which in turn affects the normal growth and metabolism of plants [2]. Pb disrupts the natural balance of ROS in plants, leading to an excessive accumulation of oxygen-free radicals and other reactive oxygen species. *D. huoshanense* is a perennial herbaceous plant belonging to the genus *Dendrobium* in the Orchidaceae family. Numerous studies have examined the impact of environmental stressors on *D. huoshanense* [3–5]. However, there is a scarcity of studies regarding the heavy metal resistance of *D. huoshanense*, especially in terms of its detoxification mechanism and resistance to Pb enrichment. Using the label-free protein quantitative approach, the expression of differently expressed proteins in *D. huoshanense* was investigated under Pb stress, which could be helpful for functional studies on its resistance to heavy metal stress.

## Data description

The tissue culture seedlings originated from the Plant Cell Engineering Center at West Anhui University (Luan, China). All the materials used for experiments were cultured from *D. huoshanense* seeds picked last year. After being sown on the medium for about 25 days, the seeds change from light yellow to green. After being cultured for 30 days, the seeds were transferred to the germination medium for further cultivation. A cycle of 40 days was used for subculture. The seedlings used in this experiment were subcultured for three generations. The materials selected were subcultured for three generations. On day 1, we sprayed the tissue culture seedlings with the prepared 200 mg/L Pb(NO_3_)_2_. A control was used with the same amount of water. Sampling was performed on days 7 and 15, respectively. Each treatment group and control group had three biological replicates. We collected leaves from the annual plants to obtain three biological replicates. The extraction of total protein from the leaves was conducted using the procedures described in previous studies [6, 7]. The protein concentrations of samples from different groups were determined using the Bradford method. An appropriate amount of SDT protein lysis solution (4% SDS, 10 mM DTT, and 100 mM TEAB) was added to the sample. Shake and mix the solution, then sonicate in an ice-water bath for 5 min. The system was reacted at 95 °C for 8 min and then centrifuged at 12,000 g for 15 min at 4 °C. 10 mM DTT was added to the supernatant and reacted at 56 °C for 1 h. A sufficient amount of IAM was then added, and the reaction was carried out for 1 h at room temperature in the dark. Pre-cooled acetone was added to the reaction solution. The reaction solution was precipitated at−20 °C for at least 2 h and then centrifuged at 12,000 g for 15 min to collect the precipitate. 1 mL of precooled acetone was added to the precipitation, resuspended, and washed. The solution was centrifuged at 12,000 g for 15 min, and the precipitate was collected and dried. For the preparation of the loading solution, add protein solubilizing solution (8 M urea, 100 mM TEAB, pH = 8.5) to dissolve the precipitation.The protein lysate, trypsin, and 100 mM Triethylammonium bicarbonate (TEAB) buffer were well combined and subjected to digestion at a temperature of 37 °C for a duration of 4 h. Subsequently, trypsin and CaCl_2_ were incorporated for overnight digestion. Formic acid was used to adjust the pH to less than 3. After mixing, the solution is placed at room temperature and centrifuged at 12,000 g for 5 min. The supernatant was slowly passed through the C18 desalting column and then washed three times with a cleaning solution (0.1% formic acid, 3% acetonitrile). The filtrate was collected and freeze-dried. One µg of solution was injected for liquid quality testing.

Chromatographic analysis refers to the previous method [8]. The EASY-nLC™ 1200 nanoscale UHPLC system was employed, and the mass spectrometry analysis was performed on a Q Exactive™ HF-X mass spectrometer equipped with a Nanospray Flex™ (ESI) ion source. The ion spray voltage is set to 2.1 kilovolts, while maintaining the ion transfer tube temperature at 320 °C. The mass spectrometer data was acquired based on the data-dependent acquisition mode. The mass spectrometer has a complete scanning range of m/z 350–1500, and the first-level mass spectrometer has a resolution of 60,000. High-energy collision fragmentation is carried out using the forty precursor ions that have the greatest ion intensity during the full scan. The high-energy collision dissociation (HCD) method was used for fragmentation, and secondary mass spectrometry with a 15,000 resolution setting was used for detection. The collision energy for the peptide fragmentation was adjusted to 27%, and the threshold intensity was set to 2.2 × 10^4^. The dynamic exclusion range was set to 20 s in order to generate raw data (.raw) for mass spectrometry detection. Protein library searches were conducted using Proteome Discoverer 2.2 (Thermo, USA). Peptide spectrum matches (PSMs) with a credibility of over 99% are considered trustworthy. Similarly, proteins that have at least one unique peptide are also considered credible. We retain only peptides and proteins with credible spectrums and perform false discovery rate (FDR) verification and removal. Peptides and proteins exhibit a FDR of over 1%. We conducted statistical analysis on the protein quantification using the T-test method. Up-regulated expression proteins were identified when FC ≥ 4.0 and p-value ≤ 0.05. Down-regulated expression proteins were identified when FC ≤ 0.25 and p-value ≤ 0.05. The interproscan software was utilized for GO and IPR functional annotation, encompassing the Pfam, PRINTS, ProDom, SMART, ProSite, and PANTHER databases (Table 1). Furthermore, functional protein pathway and family analyses were conducted on the identified proteins using the COG and KEGG databases [9–14]. Volcano plot analysis, cluster heat map analysis, subcellular localization analysis, and pathway enrichment analysis of the differentially expressed proteins were conducted using the GO, IPR, and KEGG databases [15–21]. The raw data for mass spectrometry were deposited at ProteomeXchange database [22].

**Table 1:** Overview of data files/datasets

Label	Name of data file/data set	File types (file extension)	Data repository and identifier (DOI or accession number)
Data file 1	COG annotation results of all proteins under Pb stress	EXCEL file (.XLS)	Figshare (10.6084/m9.figshare.24720894.v1) [9]
Data file 2	GO annotation results of all proteins under Pb stress	EXCEL file (.XLS)	Figshare (10.6084/m9.figshare.24720987.v1) [10]
Data file 3	IPR annotation results of all proteins under Pb stress	EXCEL file (.XLS)	Figshare (10.6084/m9.figshare.24721011.v1) [11]
Data file 4	KEGG annotation results of all proteins under Pb stress	EXCEL file (.XLS)	Figshare (10.6084/m9.figshare.24721023.v1) [12]
Data file 5	Subcellular localization results of differential proteins under Pb stress	EXCEL file (.XLS)	Figshare (10.6084/m9.figshare.24721038.v1) [13]
Data file 6	Quantitative results for all identified proteins under Pb stress	EXCEL file (.XLS)	Figshare (10.6084/m9.figshare.24721050.v1) [14]
Data file 7	Significantly different protein analysis results under Pb stress	EXCEL file (.XLS)	Figshare (10.6084/m9.figshare.24721074.v1) [15]
Data file 8	Heat map analysis of significantly different proteins under Pb stress	EXCEL file (.XLS)	Figshare (10.6084/m9.figshare.24721089.v1) [16]
Data file 9	GO enrichment of up- and down-regulated proteins under Pb stress	EXCEL file (.XLS)	Figshare (10.6084/m9.figshare.24721095.v1) [17]
Data file 10	KEGG enrichment of up- and down-regulated proteins under Pb stress	EXCEL file (.XLS)	Figshare (10.6084/m9.figshare.24721107.v1) [18]
Data file 11	IPR enrichment proteins under Pb stress	EXCEL file (.XLS)	Figshare (10.6084/m9.figshare.24721110.v1) [19]
Data file 12	Subcellular localization of differential proteins under Pb stress	EXCEL file (.XLS)	Figshare (10.6084/m9.figshare.24721119.v1) [20]
Data file 13	All protein sequences identified	FASTA file (.FA)	Figshare (10.6084/m9.figshare.24721149.v1) [21]
Data set 1	Raw data files related to the proteomics project	XML file (.XML)	ProteomeXchange (https://proteomecentral.proteomexchange.org/cgi/GetDataset?ID=PXD047050) [22]

## Limitations

There is a shortcoming that we only collected control and two periods of Pb treatment for the proteomics sequencing and no parallel reaction monitoring (PRM) verification. PRM quantitative detection technology is often used to verify the experimental results of high-throughput quantitative proteomics (such as iTRAQ, TMT, SILAC, and label-free), and can also perform relative or absolute quantitative analysis of target proteins. By detecting all fragment ion information within the selected precursor ion window, specific quantitative analysis of target proteins or peptides in complex samples can be performed.

## Data Availability

No datasets were generated or analysed during the current study.

## Abbreviations

TEAB: Triethylammonium bicarbonate
HCD: High-energy collision dissociation
PRM: Parallel reaction monitoring
